# When an Atrial Septal Defect Is No Longer Silent: A Case Report of a Large Symptomatic Defect Presenting in Adulthood

**DOI:** 10.7759/cureus.109183

**Published:** 2026-05-19

**Authors:** Joshua D Greendyk, Afif Hossain, Vinesh R Jonnala, William E Allen

**Affiliations:** 1 Department of Medicine, Rutgers University New Jersey Medical School, Newark, USA; 2 Department of Medicine, Boston Medical Center, Boston, USA

**Keywords:** adult congenital heart disease (achd), asd (atrial septal defect), ostium secundum atrial septal defect, pulmonary hypertension, transesophageal echocardiogram, transthoracic echocardiogram

## Abstract

Atrial septal defects (ASDs) are among the most common forms of congenital heart disease diagnosed in adulthood. Ostium secundum defects represent the majority of cases and may remain clinically silent for decades. Delayed diagnosis can lead to progressive right-sided cardiac chamber dilation, pulmonary hypertension, arrhythmias, and heart failure. The choice between percutaneous and surgical closure depends on anatomical characteristics such as defect size and the adequacy of surrounding septal rims.

We report the case of a 49-year-old woman with hypertension, type 2 diabetes mellitus, and hyperlipidemia who presented with one week of substernal chest pain, palpitations, and progressive dyspnea on exertion consistent with New York Heart Association (NYHA) functional class III limitation. Physical examination revealed a holosystolic murmur best heard along the tricuspid area with inspiratory accentuation. Transthoracic echocardiography identified a large secundum ASD measuring 35 mm × 22 mm with elevated pulmonary artery systolic pressure and right-sided chamber dilation. Transesophageal echocardiography confirmed a large defect with minimal aortic rim and limited atrioventricular valve rim, precluding percutaneous closure. Cardiac catheterization demonstrated bidirectional shunting with a Qp/Qs ratio of 2.65 and normal pulmonary vascular resistance. The patient subsequently underwent successful surgical ASD closure using a bovine pericardial patch. Her postoperative course was uncomplicated, with complete resolution of symptoms and normalization of pulmonary artery pressures on follow-up echocardiography six months later.

This case highlights the potential for large ASDs to remain undiagnosed until adulthood, when progressive right-sided cardiac remodeling leads to symptomatic disease. Early detection through timely echocardiographic evaluation is critical to facilitate timely intervention and may expand eligibility for less invasive percutaneous repair. Prompt recognition and closure of hemodynamically significant ASDs can prevent irreversible cardiopulmonary complications and significantly improve long-term outcomes.

## Introduction

Atrial septal defects (ASDs) are among the most common types of adult congenital heart disease (ACHD), accounting for 25%-30% of newly diagnosed adults with congenital lesions [1]. ASDs are characterized by an abnormal defect in the interatrial septum, resulting in a left-to-right shunt and causing oxygenated blood to flow from the left atrium into the right atrium and pulmonary circulation [2,3]. If diagnosis and treatment are delayed, cardiac remodeling can result in dilation of the right-sided cardiac chambers secondary to chronic volume overload from left-to-right shunting, contingent on shunt size and ventricular compliance [4]. ASD classification depends on the nature of the embryologic defect and anatomic location.

The four major types of ASDs include ostium secundum, ostium primum, sinus venosus, and coronary sinus defects. Ostium secundum defects account for 60%-75% of all ASDs and are characterized by an abnormal communication at the fossa ovalis [1]. These defects occur when there is increased reabsorption of the septum primum in the atrial roof or when the septum secundum fails to occlude the ostium secundum. Ostium primum defects occur when there is failure of the septum primum to fuse with the endocardial cushions [5]. Sinus venosus defects do not involve the true membranous septum but rather occur when either the superior or inferior vena cava overrides both atria [5]. Coronary sinus defects occur when there is a defect between the left atrium and the coronary sinus, creating a communication between both atria [5].

Patients with ASDs often remain asymptomatic into adulthood, contributing to delayed diagnosis and more advanced disease progression, sometimes necessitating surgical intervention [5]. Undetected ASDs can lead to complications, including right ventricular failure, paradoxical embolization, atrial arrhythmias, and pulmonary arterial hypertension (PAH), potentially culminating in Eisenmenger syndrome [6,7]. Furthermore, studies have also reported a higher prevalence of incomplete right bundle branch block (RBBB) in patients with ASD [8].

Adults with untreated ASDs are at risk for long-term complications irrespective of symptoms and frequently present later in adulthood. Specifically, any hemodynamically significant shunt markedly increases the risk of dilation of right-sided cardiac structures [2]. Delayed closure of hemodynamically significant ASDs may preclude percutaneous repair and necessitate surgical closure due to chamber and subvalvular apparatus dilation, causing mitral and/or tricuspid valve regurgitation [9]. ASDs can be surgically repaired with bovine pericardial patches, which integrate well into the atrial septum with minimal complications [10].

This case is clinically notable for the unusually late presentation of a large, hemodynamically significant ASD in adulthood after a prolonged asymptomatic period. The defect’s size and resultant right heart volume overload created management complexity, including considerations regarding the timing and modality of closure (percutaneous vs surgical) and the potential for irreversible pulmonary vascular changes. This delayed yet symptomatic presentation underscores the importance of recognizing subtle progression in ACHD and the impact of defect size on therapeutic decision-making.

## Case presentation

A 49-year-old woman with a past medical history notable for hypertension, type 2 diabetes mellitus on metformin, hyperlipidemia, and gastroesophageal reflux disease presented with one week of substernal chest pain. She described sharp, intermittent chest pain associated with palpitations lasting for hours. The pain radiated to her back and was worsened by exertion. She also reported dyspnea on exertion, one-pillow orthopnea, and paroxysmal nocturnal dyspnea, but denied lower extremity edema. These symptoms severely limited her daily activities. Her vital signs on presentation were unremarkable: she was afebrile at 97.6°F, heart rate was 66 beats per minute, blood pressure was 145/71 mmHg, respiratory rate was 18 breaths per minute, and oxygen saturation was 100% on room air. Physical examination was notable for a grade III/VI holosystolic murmur heard best at the right upper sternal border, louder with inspiration, along with splitting of S2 at the right lower sternal border. She was admitted for further evaluation of chest pain and New York Heart Association (NYHA) class III symptoms.

Differential diagnosis included acute myocardial infarction, pulmonary hypertension, aortic dissection, congestive heart failure, atrial fibrillation, coarctation of the aorta, ASD, ventricular septal defect, cor pulmonale, tricuspid regurgitation, and pulmonic stenosis.

Investigations

Admission laboratory workup showed a normal complete blood count. The chemistry panel was notable for low magnesium (1.5 mg/dL). All other electrolytes were within normal limits. Troponin was negative on three occasions (<0.001 ng/dL), and BNP was mildly elevated at 118 pg/mL. Electrocardiography showed a normal sinus rhythm with a heart rate of 74 beats per minute, frequent premature ventricular complexes, normal axis, and incomplete RBBB (Figure 1A). Chest X-ray showed cardiomegaly with prominent pulmonary arteries (Figure 1B).

**Figure 1:**
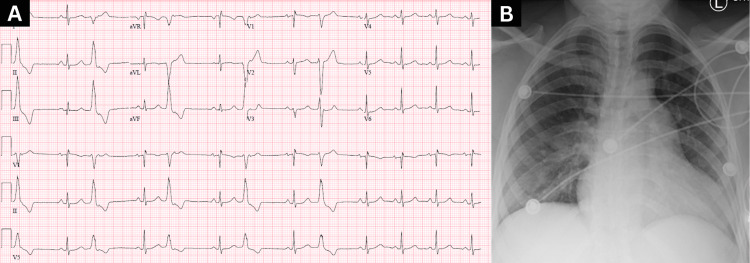
Initial electrocardiogram and chest X-ray (A) EKG showed normal sinus rhythm (heart rate 74 beats per minute) with frequent premature ventricular complexes, normal axis, and incomplete RBBB (QRS duration 90 milliseconds). (B) Chest X-ray showed cardiomegaly with a prominent left atrium and pulmonary arteries. RBBB: right bundle branch block

Computed tomography of the chest, abdomen, and pelvis showed right-sided cardiac chamber dilatation, enlarged pulmonary arteries, and engorgement of the inferior vena cava and hepatic veins, along with a large ASD, suggesting right heart failure (Figure 2).

**Figure 2:**
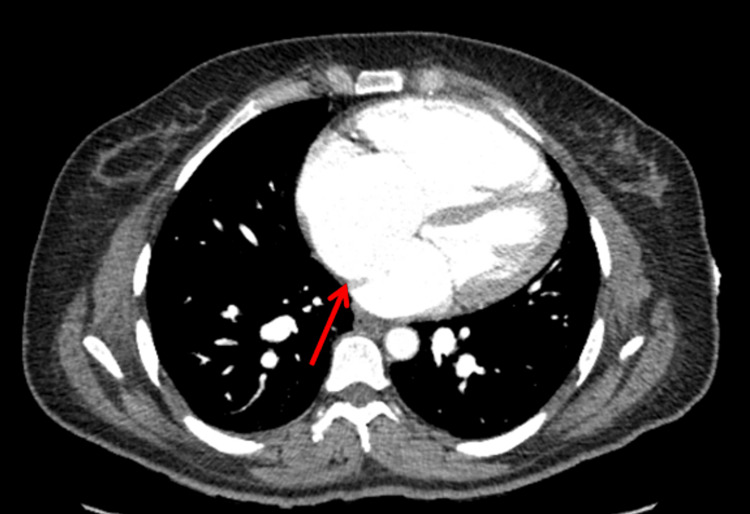
Computed tomographic angiography of the chest Computed tomographic angiography of the chest showing a large ASD, dilated right atrium and right ventricle, with signs of right ventricular pressure overload. ASD: atrial septal defect

Imaging studies were negative for pulmonary embolism or any acute aortic syndrome, such as intramural hematoma, aortic dissection, aneurysm, or rupture. Transesophageal echocardiography (TEE) showed a normal ejection fraction of 60%, an elevated pulmonary artery systolic pressure of 43 mmHg, and a secundum ASD measuring 35 mm × 22 mm (Figure 3A, 3B, 3E). A pulmonary-to-systemic shunt fraction of 1.1-1.2 was measured. However, on right heart catheterization, the Qp/Qs ratio was measured at 2.65 with normal pulmonary vascular resistance (1.04 Wood units). A subsequent TEE showed an ASD measuring 32 mm at its largest diameter with minimal to no aortic rim and a limited 2-3 mm atrioventricular valve rim (Figure 3C, 3D, 3F). The discrepancy between the near-normal echocardiographic Qp/Qs ratio (1.1-1.2) and the higher catheterization-derived Qp/Qs ratio (2.65) is most consistent with underestimation of shunt magnitude on Doppler-based echocardiography due to technical and flow-related limitations, with right heart catheterization providing the more accurate hemodynamic assessment. The minor discrepancy in shunt quantification between the two sequential TEEs is most likely attributable to differences in hemodynamic conditions and imaging technique rather than a true interval change in defect size.

**Figure 3:**
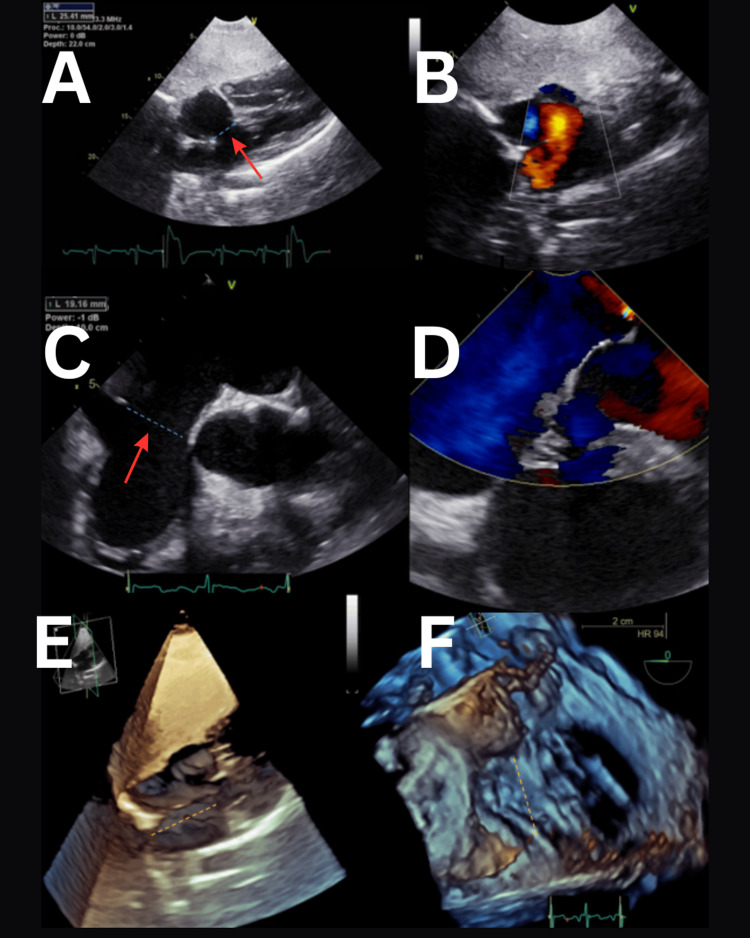
Transesophageal echocardiogram The TEE demonstrated a normal left ventricular ejection fraction with preserved systolic function, alongside severe right atrial dilation and right ventricular enlargement. (A) Subcostal TTE view showing a large secundum ASD measuring 25.4 mm (blue dashed line, red arrow) with minimal atrioventricular rim. (B) Color Doppler in the same TTE view demonstrates bidirectional flow across the ASD. (C) Mid-esophageal right ventricular inflow-outflow TEE view showing the ASD measuring 19.2 mm (blue dashed line, red arrow) with minimal aortic rim. (D) Color Doppler in the same TEE view demonstrates bidirectional flow. (E) Three-dimensional TTE reconstruction (subcostal view) outlining the ASD (yellow dashed line). (F) Three-dimensional TEE reconstruction (mid-esophageal inflow-outflow view) outlining the ASD (yellow dashed line). TEE: transesophageal echocardiography; ASD: atrial septal defect; TTE: transthoracic echocardiogram

Management

Given the large size of the ASD and minimal aortic rim, the patient’s lesion was not amenable to percutaneous closure. Therefore, she was evaluated by cardiothoracic surgery for surgical ASD repair. Prior to surgical repair, the patient underwent right and left heart catheterization, which showed no significant coronary artery disease, bidirectional flow across the ASD, a Qp/Qs ratio of 2.65, and normal pulmonary vascular resistance (1.04 Wood units). Two months after her initial presentation, she underwent uncomplicated surgical repair of the ASD with a Synovis® bovine pericardial patch. More specifically, a 5 cm × 5 cm teardrop-shaped piece of bovine pericardium was cut to fit over the ASD. Following completion of the sternotomy and repair, the patient was transferred to the cardiothoracic intensive care unit for further monitoring. There were no post-surgical complications other than a mild drop in hemoglobin, which responded appropriately to blood transfusion. She was evaluated by physical therapy, which reported improved endurance and functional activity compared with her preoperative status. She was discharged after one week on full-dose aspirin (324 mg), metoprolol tartrate, and atorvastatin with resolution of symptoms. Repeat transthoracic echocardiography six months postoperatively showed a patent surgically repaired ASD with an intact Synovis® bovine patch and normalized pulmonary artery systolic pressure of 23 mmHg (Figure 4).

**Figure 4:**
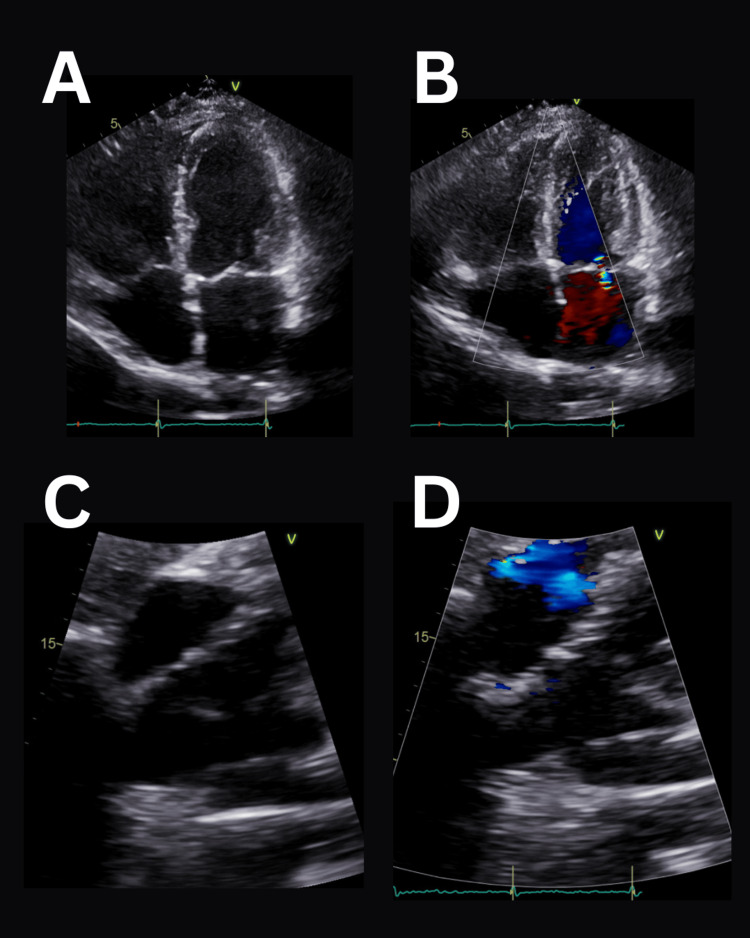
Transthoracic echocardiogram TTE in the apical four-chamber view showing: (A) intact interatrial septum status post repair of a large secundum ASD with a Synovis® bovine patch. (B) No PFO or ASD detected by color flow in the same view as A. (C) Subcostal view showing intact interatrial septum status post repair of a large secundum ASD with a Synovis® bovine patch. (D) No PFO or ASD was detected by color Doppler in the same view as C. Mild mitral regurgitation, mild right atrial dilatation, and a hypermobile interatrial septum were also present. Unfortunately, no agitated saline was injected to assess for residual intracardiac shunt. ASD: atrial septal defect; PFO: patent foramen ovale; TTE: transthoracic echocardiogram

## Discussion

Recently, significant advances have been made in facilitating earlier detection of ASDs. However, ASDs remain one of the most underdiagnosed forms of ACHD [5]. ASDs may not be diagnosed until adulthood because of minimal symptoms and anatomical heterogeneity [11]. Incidentally detected ASDs typically do not require closure if they are small and without evidence of hemodynamic compromise or cyanotic heart disease. Indications for closure often arise from right heart strain or right-to-left shunting, causing hypoxemia [12]. If early diagnosis is missed, patients can develop progressive hemodynamic compromise later in life. Hemodynamic significance is commonly assessed by the Qp/Qs ratio, which represents the ratio of pulmonary to systemic blood flow. Previously, a Qp/Qs ratio greater than 1.5:1 was considered an indication for closure, but more recent European Society of Cardiology guidelines indicate that significant ASDs are those with signs of right ventricular volume overload rather than being defined by Qp/Qs ratio alone [3]. Furthermore, the longer an ASD goes undiagnosed, the greater the likelihood of paradoxical emboli causing stroke [13]. Therefore, patients with hemodynamically significant ASDs or impending hemodynamic compromise are ideal candidates for ASD closure.

As in our case, patients with undetected ASDs often do not develop symptoms until later in life. If left uncorrected, patients with ASD can develop worsening hemodynamic compromise as well as valvular disease. Specifically, patients with large, uncorrected ASDs presenting later in adulthood often require additional repair of the mitral and/or tricuspid valves due to loss of valvular coaptation [14]. Long-term complications of ASDs include arrhythmias, paradoxical embolism, stroke, residual defects, right heart failure, and, most importantly, PAH [6,7,15]. Choosing between transcutaneous and surgical repair depends on multiple factors, including defect size, defect type, and the presence or absence of underlying heart or lung disease. Surgical closure is preferable for ASDs greater than 38 mm or those with deficient rims [16]. Although mortality rates are similar across both treatment options, surgical closure is associated with more early complications as well as longer hospitalization [17]. Therefore, early detection and repair of ASDs can greatly improve patient outcomes by mitigating the extent of adverse cardiac remodeling.

Adult ASDs often present with delayed symptoms and require a high index of clinical suspicion for diagnosis. Thus, timely transthoracic echocardiography (TTE) promotes earlier detection, medical management, and surgical or percutaneous intervention. In addition to proactive screening, an emphasis on public education regarding the symptoms, treatment options, and sequelae of ASDs is necessary. Interestingly, patients with unrepaired ASD also have an increased risk of psychiatric disorders [18]. A significant proportion of ACHD patients are lost to follow-up, leading to increased healthcare costs, associated morbidity, and ultimately progression of preventable complications [19]. Unfortunately, upwards of 80% of ACHD patients lack adequate cardiac specialist follow-up. Poor follow-up potentiates complications after ASD diagnosis and closure, including arrhythmias, stroke, failure of shunt closure, pulmonary venous congestion, left atrial hypertension, and progressive PAH [15,17]. Echocardiographic screening is a cost-effective approach shown to reduce ACHD progression and hospital readmission rates, thereby improving patient outcomes [19]. Therefore, increased echocardiographic screening and patient education are two key factors in promoting early detection and repair of ASDs.

Surgical options for ASD closure include median sternotomy, minimally invasive right mini-thoracotomy, and robot-assisted repair [11]. Sternotomy remains the standard approach for large or anatomically complex defects, offering excellent exposure and reliability at the cost of increased invasiveness and recovery time [11]. Minimally invasive and robotic approaches provide improved cosmesis and faster recovery but are best suited for smaller, uncomplicated defects with adequate rims and require specialized expertise. In this patient, the defect’s size and rim deficiency favored conventional sternotomy to ensure procedural safety and long-term efficacy. This approach provided optimal exposure for precise defect visualization and durable closure, particularly given the anatomical complexity and risk of device instability with percutaneous options.

## Conclusions

ASDs remain among the most common congenital heart lesions identified in adulthood and are frequently diagnosed late, often after the development of right-sided chamber enlargement and progressive symptoms. This case highlights how delayed recognition can coincide with advanced anatomic features, namely, a large secundum defect with deficient aortic and atrioventricular rims, which ultimately limit the feasibility of percutaneous device closure.

In this patient, the unfavorable rim anatomy and large defect size necessitated surgical repair, which resulted in straightforward and successful closure using a bovine pericardial patch with an uncomplicated postoperative course. This outcome underscores the importance of detailed anatomic assessment in guiding the appropriate intervention strategy and demonstrates that, when percutaneous options are not anatomically suitable, surgical repair remains a safe and highly effective definitive therapy. Earlier identification of ASDs before progression to complex morphology may expand eligibility for transcatheter closure and potentially reduce procedural invasiveness and long-term morbidity.
